# Functional and Flow Cytometric Analysis of Buffalo Cryopreserved Spermatozoa: Comparison of Different Breeds and Incubation Times

**DOI:** 10.22074/IJFS.2021.521116.1057

**Published:** 2021-10-16

**Authors:** Tohid Rezaei Topraggaleh, Mustafa Numan Bucak, Maryam Shahverdi, Yegane Koohestani, Ali Furkan Batur, Pegah Rahimizadeh, Pinar Ili, Murat Gul, Amir Mahdi Ashrafzade, Asghar Kazem-Allilo, Mustafa Garip, Abdolhossein Shahverdi

**Affiliations:** 1 Department of Anatomical Sciences, Faculty of Medicine, Urmia University of Medical Sciences, Urmia, Iran; 2 Department of Reproduction and Artificial Insemination, Faculty of Veterinary Medicine, Selcuk University, Konya, Turkey; 3 Department of Embryology, Reproductive Biomedicine Research Center, Royan Institute for Reproductive Biomedicine, ACECR, Tehran, Iran; 4 Department of Anatomical Sciences, Faculty of Medicine, Guilan University of Medical Sciences, Rasht, Iran; 5 Department of Urology, Selcuk University School of Medicine, Konya, Turkey; 6 Department of Medical Services and Techniques, Denizli Vocational School of Health Services, Pamukkale University, Denizli, Turkey; 7 Department of Genetics, Reproductive Biomedicine Research Center, Royan Institute for Reproductive Biomedicine, ACECR, Tehran, Iran; 8 Buffalo Breeding and Training Extension Center, Jabal, Urmia, Iran; 9 Department of Animal Science, Faculty of Veterinary Medicine, Selcuk University, Konya, Turkey; 10 Reproductive Epidemiology Research Center, Royan Institute for Reproductive Biomedicine, ACECR, Tehran, Iran

**Keywords:** Buffalo, Flow Cytometric Analysis, Iranian Azari Buffalo Breed, Italian Mediterranean Buffalo Breed, Sperm Cryopreservation

## Abstract

**Background:**

The purpose of this research was to compare the functional parameters of frozen-thawed Iranian Azari buffalo spermatozoa with imported semen samples of Italian Mediterranean buffalo (IMB) after the thawing process and 4 hours of incubation.

**Materials and Methods:**

In this experimental study, a total of twenty-four ejaculates from four Iranian Azari buffalo bulls were collected. Semen samples were diluted in AndroMed extender at a concentration of 50×106 spermatozoa/ ml. The diluted samples were filled in 0.5 ml straws and were frozen in a programmable freezer. For imported semen samples, twenty-four samples of four IMB were used, which were diluted in AndroMed extender and frozen by the same procedure. Frozen-thawed sperm motion patterns, mitochondrial activity, membrane integrity, DNA integrity, reactive oxygen species (ROS), and apoptosis status were evaluated immediately after thawing and 4 hours of incubation.

**Results:**

Post-thawed sperm motility, progressive motility (PM), mitochondrial activity, membrane integrity were significantly higher in imported semen samples in compare with Iranian Azari buffalo. After 4 hours of incubation, sperm velocity patterns were superior in Iranian Azari semen samples. Moreover, the percentage of sperm cells with un-damaged DNA was higher in Iranian semen samples compared to imported samples at the time 0 of incubation. Following 4 hours of incubation, a significant increase in intracellular ROS level leads to reduced membrane integrity, mitochondrial activity, and DNA integrity in both buffalo breeds. At time 4, Iranian samples showed significantly lower apoptosis and higher dead spermatozoa compared to imported semen samples.

**Conclusion:**

Our study showed that the post-thawed quality of Iranian Azari buffalo semen was comparable with imported samples after 4 hours of incubation. Further investigations are recommended to assess the *in vitr*o and *in vivo* fertility rate of both buffalo breeds.

## Introduction

The current population of the buffalo is estimated to be around 200 million worldwide. Approximately 204,000 head of buffalo population is bred in Iran, where it provides about 2.8% and 2.5% of Iran’s total milk and meat production, respectively (1). Some characteristics of the buffalo, including the production of high-quality milk, high adaptability to harsh climate conditions, high resistance to diseases, ability to consume the low-quality forage, as well as long productive life, made this animal valuable livestock (2, 3). However, a little consideration has been paid for the buffalo’s breeding programs in Iran regarding improving their milk and meat production.

Genetic improvement programs of buffalo received considerable attention in Italy (4). The Italian Mediterranean buffalo (IMB) is considered the best buffalo in the world, which has the highest high-fat milk production (average of 8.72 kg for Italian compared to 5.71 for Iranian Azari buffaloes during lactation period) (1, 5, 6). Moreover, it is the only breed of buffalo that has gone through a breeding selection program. The progeny test has also been accomplished to select high genetic value (high milk and meat production) bulls that are transported into semen collection centers for sperm freezing and artificial insemination (AI) (7).

Thanks to the progress of AI, it has been made possible to rapid improvement of genetic material through the propagation of desired genes from high genetic merit animals (8). The benefits of AI procedure have also been doubled by the successful freezing of the semen samples without comprising sperm quality and reducing fertilizing capability (9). Although, the fertility rate of post-thawed buffalo sperm under the field condition is poor (30% frozen-thawed vs. 60% fresh), and farmers are reluctant to breed buffalos by using AI procedure (9, 10).

Low fertility rate following AI procedure is another challenge. It may be due to sperm susceptibility to cryopreservation associated damages as well as female factors such as variable estrus length and estrus detection difficulties (11). Therefore, insemination timing and frozen-thawed spermatozoa quality play a prominent role in achieving desired results. In most previous studies, sperm quality was evaluated immediately after thawing (12-14). It seems that the post-thawed spermatozoa incubation for longer periods can broaden our understanding of the spermatozoa fertilizing capability. Several *in vitro* assessments have been developed for predicting the fertility potential of cryopreserved bull semen in AI procedure. Conventional semen assessments fail to detect some functional sperm impairments which are responsible for low fertility rate following AI procedure (15). Here, we aimed to evaluate motion characteristics, mitochondrial activity, membrane integrity, reactive oxygen species (ROS), DNA integrity, and apoptosis status of Iranian Azari and IMB semen samples during 4 hours of incubation.

## Materials and Methods

This study was approved by Institutional Ethics Committee of Royan Institute (No: 90000115).

### Semen collection and cryopreservation

The semen collection of Iranian Azari buffaloes was performed in Buffalo Breeding Center, Urmia, Iran. Twenty-four ejaculates from four mature buffalo bulls (*Bubalus bubalis*), were collected. The semen samples with the volume of 2-6 ml, progressive motility (PM) 70%, and the concentration of more than 1×109 spermatozoa/ml were enrolled in the study (12). The sperm concentration was determined by a digital photometer (IMV, France) and were diluted in AndroMed extender at a concentration of 50×10^6^ spermatozoa/ml according to the manufacturer's instructions (Mintube, Germany) (16). The diluted samples were cooled to 4°C in 2 hours, and were left to equilibrate at 4°C for 2 hours. Then, samples were filled in 0.5 ml straws and were frozen in a cell freezer according to digit-cool (IMV Technologies, France) standard curve for bull semen (5°C/minutes from+4°C to- 10°C;-40°C/minutes from-10°C to-100°C and-20°C/minutes from-100°C to 140°C). Also, twenty-four samples of four IMB were used, which were diluted in AndroMed extender and frozen by the same procedure. The frozen samples were thawed at 37°C for 30 seconds. Half of the samples were immediately analyzed after thawing (time 0), while the remainder was incubated at 37°C for 4 hours in a 5% CO_2_ and analyzed after incubation (time 4).

All assessments were performed at the Department of Embryology, Reproductive Biomedicine Research Center, Royan Institute, ACECR, Tehran, Iran.

### Motion parameters

The motion parameters of Iranian Azari and imported frozen-thawed buffalo sperm with ~ 50×106 spermatozoa/ml concentration were analyzed by a computer-aided sperm analyzer (CASA, Sperm Class Analyzer, version 5, Microptic, Spain). The CASA system was adjusted for bull semen, according to Topraggaleh et al. (12). A 10 µl aliquot of semen sample was placed on a pre-warmed SpermTrack ® chamber (Proiser, Spain) and sperm motion characteristics, including total motility (TM, %), PM (%), curvilinear velocity (VCL, µm/seconds), straight line velocity (VSL, µm/seconds), average path velocity (VAP, µm/seconds), straightness (STR, %), linearity (LIN, %), amplitude of lateral head displacement (ALH, µm/seconds), wobble (WOB, %), and beat cross frequency (BCF, Hz) were analyzed in 5 randomly-selected microscopic fields with approximately 500 spermatozoa.

### Membrane integrity

The sperm functional membrane integrity was assessed by hypo-osmotic swelling (HOS) test. Briefly, 50 μl of frozen-thawed samples were diluted in 500 μl of HOS solution [1.351 g fructose (1.05321, Merck, Germany) and 0.735 g sodium citrate dehydrate (W302600, Sigma-Aldrich, USA) were dissolved in 100 ml of distilled water; 190 mOsm/kg] and incubated for 45 minutes at 37°C in a 5% CO_2_ (17). Afterward, 10 μl of suspension was placed on a glass slide and mounted with a coverslip. A total of 200 sperm cells were analyzed under a phase-contrast microscope (Olympus BX20) at a magnification of 400×. Sperm cells with coiled or swollen tail were considered as the functional plasma membrane.

### Flow cytometry analysis

Using the FACS Calibur flow cytometer (BD Immunocytometry Systems, USA), mitochondrial activity, DNA fragmentation, intracellular ROS, and apoptosis were analyzed. Imaging was made under excitation of an argon laser at 488 nm. To exclude debris and aggregates, the sperm cell population was gated using 90° and forward-angle light scatter. The green fluorescence (intact DNA and low mitochondrial activity) was measured with FL1 detector (530 nm), while the red fluorescence [damaged DNA, propidium iodide (PI)] was measured with FL3 detector (620 nm). The fluorescence of multimeric form of JC-1 (high mitochondrial activity) and Dihydroethidium (DHE) were determined with FL2 detector (585 nm). A minimum of 10.000 sperm cells was assessed in each sample at the flow rate of 100 cells/s and analyzed by Flowing Software version 2.5.1 (Cell Imaging Core, Finland).

### Mitochondrial activity

The sperm mitochondrial activity was investigated using, JC-1 dye (T4069, Sigma-Aldrich, USA). Briefly, post-thawed semen specimens were centrifuged at 500 g for 5 minutes. The supernatant was removed, and cell pellets were resuspended in phosphate-buffered saline (PBS) at a final concentration of 1×106 cells/ml. Then, 1 µL of JC-1 stock solution [200 μM dissolved in DMSO (D2650, Sigma-Aldrich, USA)] was added into 1 ml of cell suspension, incubated at 37°C for 40 minutes in a dark place, and cells were finally subjected to flow cytometry (11).

### DNA integrity

Sperm DNA damage was measured according to the sperm chromatin structure assay protocol. Post-thawed semen samples were centrifuged at 500 g for 5 minutes, supernatants were discarded, and remaining cells were diluted with Tris Null EDTA buffer (150 mM NaCl, 1 mM EDTA, and 10 mM Tris at pH=7.2) at a final concentration of 5×10^6^ cells / ml. Then, 400 μl of acidic solution (0.15 M NaCl and 0.08 M HCl in 0.1% v/v Triton X-100) was added to 200 μl of diluted samples. After 30 seconds incubation, 1.2 ml of acridine orange (AO) solution [6 μg/ml AO (A8097, Sigma-Aldrich, USA), 0.1 M citric acid, 1 mM EDTA, 0.2 M Na_2_HPO_4_, and 0.15 M NaCl at pH=6.0] was added. Finally, cells were subjected to flow cytometry after 30 minutes incubation (18).

### Reactive oxygen species

Intracellular ROS was determined by DHE. In brief, postthawed semen samples were resuspended with PBS at a concentration of 1×106 cells/ml. An aliquot of 10 μl of DHE stock solution (1.25 mM, D 7008, Sigma-Aldrich, USA) was added into 1 ml of diluted semen samples, incubated at 25°C for 20 minutes, and subjected to flow cytometry (19).

### Apoptosis status

Apoptosis status of frozen-thawed spermatozoa was determined by the double-stained method with Annexin Vfluorescein isothiocyanate (FITC) and PI according to the manufacturer’s directions (IQP, Groningen, Netherlands). Sperm samples were washed in calcium buffer, and concentration was adjusted to 1×10^6^ cells /ml. An aliquot of 10 μl of Annexin V-FITC (0.01 mg/ml) was added to 100 μl of sperm samples and incubated for 20 minutes on ice. Then, 10 µl of PI (1 µg/ml) was mixed with sperm suspension and incubated for 10 min on ice prior to evaluation with flow cytometry. Following analysis, sperm cells were classified into three categories: i. Annexin V and PI negative considered as viable non-apoptotic cells, ii. Annexin-V positive, but PI negative marked as apoptotic cells, and iii. Positive for both Annexin-V and PI as well as negative for Annexin-V but positive for PI were regarded as dead cells (20).

### Statistical analysis

In statistical evaluation, the variance in repeated measurements was evaluated by the procedure GLM repeated measurement. Bull and origin were taken as factors. The result of different times, T0 and T4 (4 hours), were compared. A sample dependent t-test was used for two-group comparisons, and the Bonferroni test was used for multiple comparisons. All parameters were analyzed using the SPSS/PC software package (IBM SPSS Statistics Inc. version 25.0, Chicago, IL). The Statistical significance was set at P<0.05.

## Results

### Motion characteristics

Post-thawed Italian Mediterranean and Iranian Azari buffalo semen motion characteristics are displayed in Table 1. In both buffalo breeds, post-thawed sperm motion parameters, including TM, PM, VAP, VSL VCL, ALH, and BCF were significantly decreased following 4 hours of incubation. At the time 0, sperm characteristics, including TM, PM, VSL, STR, and ALH were significantly higher in Italian buffalo semen compared to Iranian samples. However, after 4 hours of incubation, statistical significant differences were not seen in TM, PM, VCL, VSL, and VAP between imported and Iranian buffalo semen samples. Iranian Azari buffalo semen showed significantly higher LIN, STR, and WOB compared to imported samples after 4 hours of incubation.

### Membrane integrity, mitochondrial activity, DNA integrity, and ROS

As shown in Table 2, post-thawed membrane integrity and mitochondrial activity were significantly decreased during 4 hours of incubation in both of the buffalo breeds. However, the percentage of cells with intracellular ROS and damaged DNA were significantly increased following incubation in both the Italian Mediterranean and Iranian Azari buffalo semen samples. Italian buffalo semen samples showed significantly higher membrane integrity and mitochondrial activity compared to Iranian buffalo samples immediately after thawing. However, the percentage of sperm cells with fragmented DNA was significantly lower in Iranian Azari samples compared to imported straws at time 0 of incubation (4.60 ± 0.16 vs. 5.58 ± 0.20). No statistically significant differences were observed in all parameters among the Italian Mediterranean and Iranian Azari buffalo straws after 4 hours of incubation.

**Table 1:** Sperm motion characteristics between native and imported semen samples during 0 and 4 hours of incubations


Variables	Iranian Azari		Italian Mediterranean	
	0 hour	4 hours	0 hour	4 hours

TM (%)	56.77 ± 2.02^aB^	33.69 ± 1.87^b^	66.27 ± 1.78^aA^	38.51 ± 2.43^b^
PM (%)	41.19 ± 2.35^aB^	19.44 ± 1.41^b^	48.54 ± 2.57^aA^	20.85 ± 1.77^b^
VCL (µm/s)	69.55 ± 3.71^a^	38.31 ± 2.40^b^	78.39 ± 2.69^a^	41.70 ± 3.10^b^
VSL (µm/s)	37.24 ± 2.13^aB^	21.26 ± 1.57^b^	44.95 ± 2.44^aA^	19.60 ± 2.46^b^
VAP (µm/s)	52.51 ± 3.17^a^	29.63 ± 2.47^b^	59.70 ± 2.79^a^	29.59 ± 3.59^b^
LIN (%)	53.28 ± 0.72	54.42 ± 1.40^A^	56.85 ± 2.12^a^	42.20 ± 3.54^bB^
STR (%)	71.26 ± 0.50^B^	72.98 ± 1.16^A^	74.70 ± 1.34^aA^	63.63 ± 1.68^bB^
WOB (%)	74.75 ± 0.89	74.81 ± 1.93^A^	75.52 ± 1.62^a^	64.29 ± 4.42^bB^
ALH (µm)	2.60 ± 0.07^aB^	1.72 ± 0.02^bA^	2.98 ± 0.08^aA^	1.93 ± 0.05^bB^
BCF (Hz)	7.89 ± 0.16^a^	7.47 ± 0.11^b^	7.95 ± 0.17^a^	7.22 ± 0.25^b^


Data are presented as the mean ± SE. Small letters (a, b) in same raw indicate significant differences (P<0.05) between the 0 and 4 hours of incubation times, capital letters (A and B) in same raw indicate significant differences (P<0.05) between native and imported semen samples in the same time. TM; Total motility, PM; Progressive motility, VCL; Curvilinear velocity, VSL; Straight line velocity, VAP; Average path velocity, LIN; Linearity, STR; Straightness, WOB; Wobble, ALH; Lateral head displacement, and BCF; Beat cross frequency.

**Table 2:** Sperm membrane integrity, mitochondrial activity, DNA integrity and ROS between native and imported semen samples during 0 and 4 hours of incubations


Variables	Iranian Azari		Italian Mediterranean	
	0 hour	4 hours	0 hour	4 hours

Membrane integrity (%)	66.56 ± 2.26^aB^	43.64 ± 2.50^b^	73.96 ± 1.72^aA^	47.76 ± 1.88^b^
Mitochondrial activity (%)	37.15 ± 1.90^aB^	19.38 ± 0.77^b^	42.63 ± 1.83^aA^	20.29 ± 0.92^b^
DNA fragmentation (%)	4.60 ± 0.16^bB^	6.73 ± 0.32^a^	5.58 ± 0.20^bA^	7.33 ± 0.39^a^
ROS (%)	49.72 ± 1.66^b^	63.68 ± 2.62^a^	49.65 ± 2.10^b^	65.50 ± 2.38^a^


Data are presented as the mean ± SE. Small letters (a, b) in same raw indicate significant differences (P<0.05) between the 0 and 4 hours of incubation times, capital letters (A and B) in same raw indicate significant differences (P<0.05) between native and imported semen samples in the same time. ROS; Reactive oxygen species motility.

**Table 3:** Percent of live, early apoptosis, late apoptosis and necrosis between native and imported semen samples during 0 and 4 hours of incubations


Variables	Iranian Azari		Italian Mediterranean	
	0 hour	4 hours	0 hour	4 hours

Membrane integrity (%)	59.79 ± 2.09^a^	24.98 ± 1.34^b^	55.94 ± 1.78^a^	26.38 ± 1.52^b^
Mitochondrial activity (%)	10.30 ± 0.59^b^	12.27 ± 0.60^aB^	9.77 ± 0.67^b^	16.32 ± 0.70^aA^
DNA fragmentation (%)	29.49 ± 2.22^b^	62.74 ± 1.56^aA^	34.28 ± 1.83^b^	57.28 ± 1.84^aB^


Data are presented as the mean ± SE. Small letters (a, b) in same raw indicate significant differences (P<0.05) between the 0 and 4 hours of incubation times, capital letters (A and B) in same raw indicate significant differences (P<0.05) between native and imported semen samples in the same time.

### Apoptosis status

The apoptosis status of frozen-thawed Italian Mediterranean and Iranian Azari buffalo semen is presented in Table 3. Following the incubation of semen samples, the percentage of live cells significantly decreased. Nevertheless, the percentage of dead spermatozoa, and also apoptotic spermatozoa were significantly increased during 4 hours of incubation in both buffalo semen samples. There were no significant differences in apoptosis status at time 0 between buffalo samples. However, at time 4, Iranian Azari samples showed significantly lower apoptosis and higher dead spermatozoa compared to Italian Mediterranean semen samples.

## Discussion

In order to increase the milk production potential of Iranian buffalo, the Animal Breeding Center of Iran (ABCI, Karaj, Iran) imported semen samples of high genetic merit IMB bulls. However, follow-up of the inseminated samples, rate of fertilization as well as *in vitro* assessment of frozen-thawed imported samples were not precisely evaluated. For the first time, we compared *in vitro* characteristics of frozen-thawed sperm between Italian Mediterranean and native Iranian Azari buffalos.

The present study’s data showed that sperm characteristics, including motion parameters, membrane integrity, and mitochondrial activity, were significantly higher in Italian spermatozoa compared to the Iranian Azari group. As expected, the quality of frozen-thawed spermatozoa could be influenced by the age, feeding, and housing conditions as well as environmental factors, including humidity, temperature, and day length (21, 22). Moreover, semen processing, including dilution, type of extender, equilibration, freezing, and thawing, influences the quality of frozen-thawed buffalo spermatozoa (13, 23, 24). A growing body of literature has shown that the quality of post-thawed buffalo spermatozoa has been diminished by increasing the temperature in tropical and subtropical countries during the years (21, 25, 26). Although we tried to minimize the extrinsic factors (such as feeding and housing condition, semen processing) of two study groups, the average temperature of the Iranian buffalo breeding place was higher than Italian ones during the semen collection period (February-April). Thus, the lower post-thawed quality of Iranian buffalo spermatozoa could be attributed to genetic differences between the two studied breeds as well as environmental conditions, higher temperature and lower weather humidity.

Another finding of this study was that incubation of post-thawed semen samples for 4 hours significantly decreased motility, velocity patterns, membrane integrity, and mitochondrial activity as well as increased intracellular ROS and DNA fragmentation in both of imported and native semen samples. These findings are in accordance with the results of Rastegarnia et al. (24), where incubation of post-thawed buffalo spermatozoa for 4 hours significantly decreased the sperm quality in soybean lecithin and egg yolk based extenders. In the nature, semen is deposited in the vagina near the external os of the cervix. Sperm cells may be transported to the site of fertilization in two phases. Rapid phase in which a lower quantity of sperm cells is transported to the ampulla region within a few minutes. And slow phase in which a large number of sperm cells move toward the oviduct over the 4-8 hours. This time of sperm transport to the site of fertilization is decreased to 30 minutes following AI procedure due to sperm deposition in the uterus' horns. Although assessments of sperm parameters immediately after thawing indicate sperm quality to some extent, *in vitro* incubation of thawed semen samples for longer periods of time broaden our understanding of spermatozoa's fertilizing capability. A large amount of the ROS is generated by the electron transport chain of mitochondria during the spermatozoa incubation (27). A high level of ROS, along with insufficient antioxidant defenses, leads to oxidative stress in spermatozoa (28). Excess production of ROS induces structural and biochemical alteration, including depletion of ATP, DNA fragmentation, and lipid peroxidation in spermatozoa (29). Therefore, decreased sperm motility, membrane integrity, mitochondrial activity, and DNA integrity are likely to be related to increased intracellular ROS levels during incubation.

Frozen in different semen collection condition was one of the major limitations of this study. Although we tried to minimize the differences between the semen processing and freezing of the both buffalo bulls, intrinsic factors like the genetics of the bulls could also affect the quality of frozen-thawed spermatozoa. Another limitation of this study was that, the comparison of the samples was performed only by analyzing sperm *in vitro* characteristics. Pregnancy rate, as well as delivery of live offspring following insemination of both semen samples, was not evaluated in this study. The main reason behind this problem is that the farming system of buffalo in Iran is based on small holders (99%) with an average herd size of five animals (1). Since management and environmental factors could differ between the buffalo breeders, comparison of pregnancy and delivery rate following insemination of Iranian Azari and IMB samples could be challenging. Therefore, *in vivo* studies on a larger buffalo population are required to investigate the pregnancy rate following insemination of these two buffalo breeds.

## Conclusion

This study has shown that post-thawed sperm characteristics, including motility, PM, membrane integrity, and mitochondrial activity were significantly higher in Italian Mediterranean semen samples compared to Iranian Azari buffalo semen immediately after thawing. While after 4 hours of incubation, sperm velocity patterns were superior in Iranian Azari semen samples. Moreover, the percentage of sperm with intact DNA was higher in Iranian semen samples than imported samples at the time 0 of incubation. At time 4, Iranian samples showed significantly lower apoptosis and higher dead spermatozoa compared to imported semen samples. Our study indicated that the Iranian Azari buffalo semen were comparable to imported samples after 4 hours of incubation. One of the major limitations of this study was that the comparison of the Italian Mediterranean and Iranian Azari buffalo semen samples was performed only by analyzing sperm* in vitro* characteristics. Further studies are required to evaluate the *in vivo* and *in vitro* fertility rate of both buffalo breeds.
